# Predictors of survival in a cohort of patients with polymyositis and dermatomyositis: effect of corticosteroids, methotrexate and azathioprine

**DOI:** 10.1186/ar3704

**Published:** 2012-01-27

**Authors:** Elena Schiopu, Kristine Phillips, Paul M MacDonald, Leslie J Crofford, Emily C Somers

**Affiliations:** 1 Department of Internal Medicine, Division of Rheumatology, University of Michigan, 1500 East Medical Center Drive, 3918 TC, Ann Arbor, MI 48109-5358, USA; 2 Department of Community Medicine, The Lahey Clinic, 267 Boston Road, Suite 20, Billerica, MA 01862, USA; 3 Department of Internal Medicine, Division of Rheumatology, University of Kentucky, Room J503 Kentucky Clinic, Lexington, KY 40536, USA

## Abstract

**Introduction:**

The idiopathic inflammatory myopathies are rare diseases for which data regarding the natural history, response to therapies and factors affecting mortality are needed. We performed this study to examine the effects of treatment and clinical features on survival in polymyositis and dermatomyositis patients.

**Methods:**

A total of 160 consecutive patients (77 with polymyositis and 83 with dermatomyositis) seen at the University of Michigan from 1997 to 2003 were included. Medical records were abstracted for clinical, laboratory and therapeutic data, including initial steroid regimen and immunosuppressive use. State vital records were utilized to derive mortality and cause of death data. Survival was modeled by left-truncated Kaplan-Meier estimation and Cox regression.

**Results:**

The 5- and 10-year survival estimates were 77% (95% CI = 66 to 85), and 62% (95% CI = 48 to 73), respectively, and the rates were similar for polymyositis and dermatomyositis. Survival between the sexes was similar through 5 years and significantly lower thereafter for males (10-year survival: 18% male, 73% female; *P *= 0.002 for 5- to 10-year interval). The sex disparity was restricted to the polymyositis group. Increased age at diagnosis and non-Caucasian race were associated with lower survival. Intravenous versus oral corticosteroid use was associated with a higher risk of death among Caucasians (HR = 10.6, 95% CI = 2.1 to 52.8). Early survival between patients treated with methotrexate versus azathioprine was similar, but survival at 10 years was higher for the methotrexate-treated group (76% vs 52%, *P *= 0.046 for 5- to 10-year interval).

**Conclusions:**

Patients treated initially with intravenous corticosteroids had higher mortality, which was likely related to disease severity. Both methotrexate and azathioprine showed similar early survival benefits as first-line immunosuppressive drugs. Survival was higher between 5 and 10 years in the methotrexate-treated group, but could not be confirmed in multivariable modeling for the full follow-up period. Other important predictors of long-term survival included younger age, female sex and Caucasian race.

## Introduction

Polymyositis (PM) and dermatomyositis (DM) are idiopathic inflammatory myopathies (IIMs) characterized by chronic muscle inflammation leading to progressive symmetrical muscle weakness [1] and lung disease which can include interstitial lung disease (ILD) [2], respiratory muscle weakness and aspiration. A literature review estimated PM and DM prevalence as 5.1 per 100,000 (1965 to 1995), with an overall female preponderance (67%) [3]. A more recent review (1989 to 2008) found no new estimates, underscoring the need for contemporary epidemiologic data [4].

PM and DM are associated with considerable morbidity due to damage caused by both the disease and its treatment, and 5-year survival estimates vary from 60% to 80% [5-8]. IIM patients self-report significantly poorer health compared to the general population, and patients with chronic, progressive disease experience significantly greater pain. The therapeutic management of myositis is challenging. Conventional approaches include baseline measurements of muscle involvement followed by prednisone for 6 to 8 weeks [9]. Although corticosteroids often remain the initial therapeutic choice, side effects and limited efficacy require additional steroid-sparing agents. The choice of agent largely depends on the experience of the prescribing physician, and few data exist to guide choice of drugs at initial treatment stages.

Comparative effectiveness research evaluates the benefits and harms of different interventions, frequently in a "real-world" setting. Immunosuppressive agents are widely used in myositis as steroid-sparing agents, but studies contrasting the long-term effectiveness of immunosuppressive agents are lacking. We performed this study to characterize mortality outcomes among IIM patients and to evaluate predictors of mortality, including demographics, clinical variables and treatment at initial presentation.

## Materials and methods

### Patient population

A retrospective study was performed, including individuals meeting eligibility criteria, with in- or outpatient visits at the University of Michigan between January 1997 and March 2003. Patients were identified based on *International Classification of Diseases, Ninth Revision, Clinical Modification *codes for PM or DM [10]. The study population included prevalent and incident cases with relevant codes, which were validated to meet probable or definite Bohan and Peter criteria [11,12]. Patients with inclusion body myositis (IBM) or overlap with other connective tissue diseases were excluded. Medical charts were reviewed for demographic, clinical and laboratory data. Vital records for mortality and cause of death data were obtained from the Michigan Department of Community Health. This study was approved by the University of Michigan Institutional Review Board, with a waiver for informed consent.

### Clinical data

Data collected included demographics, clinical characteristics, medications, creatine kinase (CK) levels (as a surrogate for disease activity), ILD (based on findings consistent with ILD derived from pulmonary function testing, chest X-ray or high-resolution computed tomography), vital status and/or loss to follow-up with associated dates. Treatment data included initial steroid regimen categorized as none, intravenous corticosteroid pulse treatment (IV pulse), high-dose oral prednisone (≥ 60 mg/day) or lower-dose oral prednisone (< 60 mg/daily). Immunosuppressive use, including methotrexate (MTX) and azathioprine (AZA), was also documented.

### Statistical analysis

The demographic characteristics between the PM and DM groups were compared by two-sample *t*-tests, Wilcoxon rank-sum tests or χ^2 ^tests. One-way analysis of variance or Kruskal-Wallis tests were used to compare continuous measures between more than two categories. Survival analysis was performed by the Kaplan-Meier method using the left-truncated approach to adjust for survival bias. Thus we estimated the probability of survival as a function of time since diagnosis, with risk of death restricted to the observation period (that is, after January 1997). Follow-up time was calculated as time from diagnosis until the first occurrence of one of the following: death, loss to follow-up or censoring at the end of the observation period (July 2003). The equality of survival functions was calculated by logrank test. Left-truncated univariate and multivariable Cox proportional hazards regression were performed to estimate hazard ratios. Data management and analysis were performed using Stata software (StataCorp, College Station, TX, USA).

## Results

### Patient characteristics

A total of 160 consecutive patients (77 PM and 83 DM) comprised the study population. Characteristics of the PM and DM patients are outlined in Table 1. Overall, 116 (72%) of the patients were female, and the patients' mean age (± SD) at diagnosis was 48.4 ± 13.5 years. Lifetime history of cancer was similar between PM and DM patients, though the proportion of cancers diagnosed 3 years before or after diagnosis was significantly higher in the DM group (11 of 12, 91.7%) than in the PM group (5 of 13, 38.5%) (*P *= 0.01). Among patients with a history of malignancy, the types of cancers in the 13 PM patients were 5 gynecologic, 3 breast, 2 lung, 2 skin and 1 lymphoma; in the 12 DM patients, the cancer types were 3 gastrointestinal, 2 gynecologic, 2 breast, 2 skin, 2 lymphoma and 1 prostate.

**Table 1:** Demographics and baseline characteristics of patients with PM versus DM^a^

Characteristics	PM (*n *= 77)	DM (*n *= 83)	*P *value
Age at diagnosis (years)	49.7 ± 13.0	47.2 ± 13.9	0.236
Females, *n *(%)	60 (77.9)	56 (67.5)	0.139
Race, *n *(%)			0.042
White, non-Hispanic	61 (79.2)	52 (62.7)	
Black	13 (16.9)	15 (18.1)	
Hispanic	0 (0)	2 (2.4)	
Asian	0 (0)	1 (1.2)	
Other or unknown	3 (3.9)	13 (15.7)	
Median [IQR] initial CK level (IU/L)^b^	3,250 [846 to 11,698]	415 [71 to 3,000]	0.0005
Interstitial lung disease, *n *(%)	21 (27.3)	16 (19.3)	0.231
Cancer, *n *(%)			
Ever	13 (16.9)	12 (14.5)	0.673
Proportion diagnosed within ± 3 years of IIM diagnosis	5 (38.5)	11 (91.7)	0.01

^a^CK = creatine kinase; DM = dermatomyositis; IIM = idiopathic inflammatory myopathy; PM = polymyositis. ^b^Includes CK values (if available) within 3 months of IIM diagnosis. Results are expressed as means ± SD or number (%).

### Treatment

Data on the initial corticosteroid regimen was available for 110 patients (20 IV pulse, 56 high-dose oral (≥ 60 mg/day), 27 < 60 mg/day oral and 7 no steroids). An additional 50 patients were treated with steroids of unknown dose. Treatment was administered in the inpatient setting for 90% of patients on IV pulse corticosteroids, with 14% of patients receiving the high dose orally and 0% of patients taking < 60 mg/day orally. CK levels were available within 3 months of PM and DM diagnosis in 73 of the 153 patients who were treated with corticosteroids. CK levels (in IU/L) tended to be highest in the IV pulse group (median = 2,905, IQR = 681 to 11,062), followed by high-dose oral (median = 2,216, IQR = 300 to 6,488) then lower-dose oral (median = 791, IQR = 30 to 4,000), though these levels were not significantly different.

Immunosuppressive drugs were used in 147 (92%) of the patients, with the largest proportion being MTX (*n *= 75, 47%) and AZA (*n *= 51, 32%). Other immunomodulatory agents utilized were cyclophosphamide (6.3%), cyclosporine (3.1%), mycophenolate mofetil (1.3%), TNF blockers (0.6%) and intravenous immunoglobulin (1.9%). Baseline characteristics, including diagnoses of PM and DM, sex, race and age at diagnosis, were similar for patients treated with MTX or AZA. Median CK levels within 3 months of diagnosis in the 118 of 126 patients for which we had available data who received either MTX (median = 720, IQR = 109 to 2,600) or AZA (median = 232, IQR = 83 to 1,766) did not reach a statistically significant difference.

### Survival

**Figure 1:**
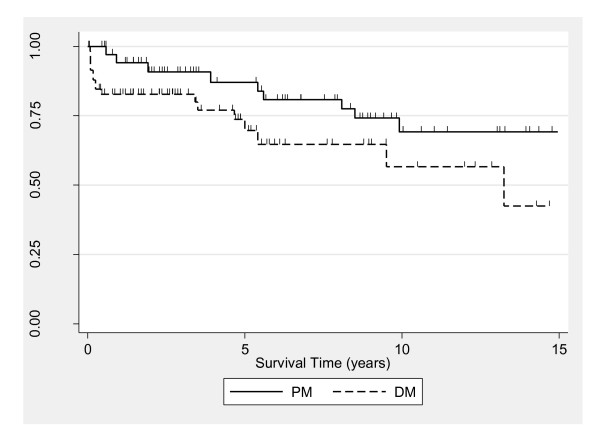
**Kaplan-Meier estimates for survival in polymyositis (PM) and dermatomyositis (DM) patients**. Each tick mark corresponds to a time of patient censoring. Survival rates were not significantly different according to diagnosis (*P *= 0.12).

The median follow-up was 4.6 years (IQR = 1.7 to 8.8). The total time at risk in the analysis, corresponding to the 160 patients, was 572.2 person-years. Overall 5- and 10-year survival for the study population were estimated to be 77% (95% CI = 66 to 85) and 62% (95% CI = 48 to 73), respectively. Rates were not statistically different between PM and DM, though they appeared higher for PM (*P *= 0.12). Survival rates for PM were 87% (95% CI = 69 to 95) at 5 years and 69% (95% CI = 49 to 83) at 10 years, and for DM the rates were 70% (95% CI = 53 to 82) at 5 years and 57% (95% CI = 34 to 74) at 10 years (Figure 1).

**Figure 2:**
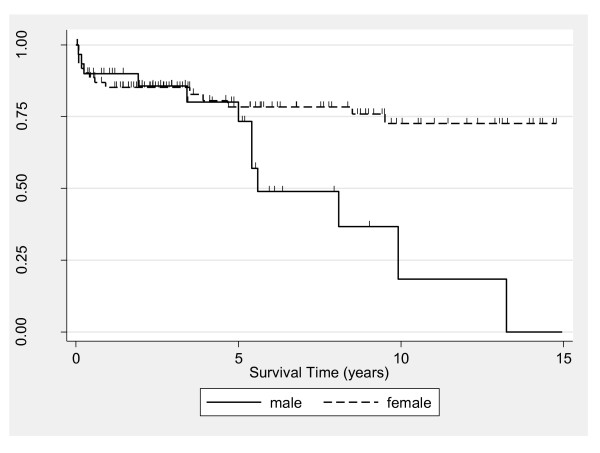
**Kaplan-Meier estimates comparing survival in patients with idiopathic inflammatory myopathy (polymyositis and dermatomyositis) by sex**. Each tick mark corresponds to a time of patient censoring. The sex disparity was most pronounced among the polymyositis subset (see text "Survival" section).

**Figure 3:**
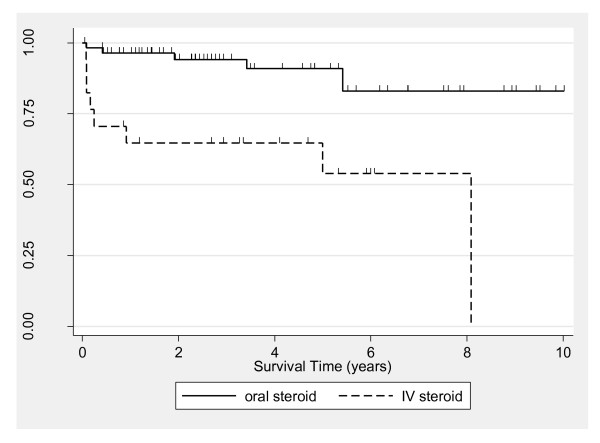
**Kaplan-Meier estimates comparing survival among idiopathic inflammatory myopathy patients according to initial corticosteroid regimen of intravenous (IV) versus oral**. Each tick mark corresponds to a time of patient censoring.

**Figure 4:**
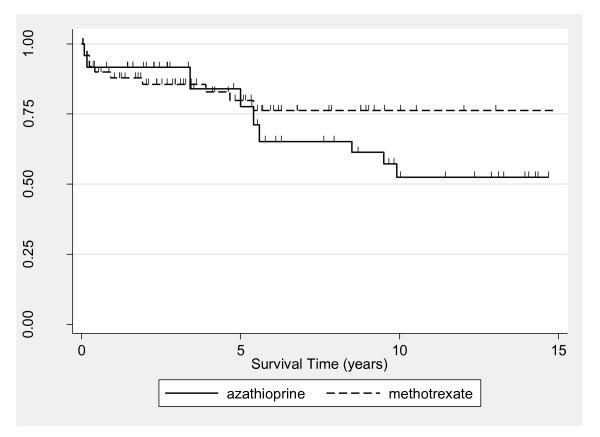
**Kaplan-Meier survival estimates, according to azathioprine versus methotrexate use**. Each tick mark corresponds to a time of patient censoring.

Although 5-year survival for the total PM and DM population was similar between males (median = 73%, 95% CI = 48 to 88) and females (median = 78%, 95% CI = 65 to 87) (*P *= 0.74), survival rates between the sexes diverged thereafter (Figure 2). The 10-year survival among males (median = 18%, 95% CI = 1 to 51) was significantly lower than among females (median = 73%, 95% CI = 57 to 83) (logrank *P *= 0.002 corresponding to the 5- to 10-year analysis time interval). Significance testing was not appropriate over the full follow-up period because of lack of conformity with the proportional hazards assumption. Further inspection utilizing Cox modeling (Table 2) revealed a significant interaction between sex and PM and DM diagnoses, with the disparity in survival by sex detected only in the PM group. Kaplan-Meier estimates adjusted for sex were similar for the PM and DM groups (data not shown), with sex-adjusted 5-year survival rates of 62% and 65%, respectively (*P *= 0.31). Kaplan-Meier estimates according to initial corticosteroid regimen indicated higher mortality for the IV versus oral group (*P *= 0.0002) (Figure 3). Five-year survival estimates were 91% (95% CI = 77 to 97) and 54% (95% CI = 25 to 76) for the oral and IV groups, respectively. When the oral corticosteroid group by was stratified dose, survival was similar between patients receiving < 60 versus ≥ 60 mg/day (data not shown). Estimates comparing survival according to type of immunosuppressive revealed similar 5-year survival for patients treated with MTX (80%, 95% CI = 64 to 89) versus AZA (78%, 95% CI = 49 to 91) (Figure 4). From 5 years onward, however, survival was higher in the MTX group. The 10-year survival was 76% for MTX (95% CI = 60 to 87) and 52% for AZA (95% CI = 28 to 72) (*P *= 0.046 for the 5- to 10-year interval).

**Table 2:** Results derived from multivariable Cox regression models^a^

	Model A: No medications included		Model B: Corticosteroid included		Model C: AZA/MTX included	
	
	HR (95% CI)	*P *value	HR (95% CI)	*P *value	HR (95% CI)	*P *value
Sex (male referent)	0.14 (0.04 to 0.58)	0.006	0.10 (0.01 to 1.27)	0.076	0.09 (0.02 to 0.52)	0.007
Age at diagnosis (years)	1.07 (1.04 to 1.11)	0.000	1.06 (1.01 to 1.12)	0.018	1.11 (1.06 to 1.16)	0.000
Race (white referent)						
Black	3.94 (1.48 to 10.46)	0.006	1.5 (0.3 to 7.4)	0.61	9.5 (2.42 to 37.09)	0.001
Other or unknown	2.03 (0.66 to 6.29)	0.22	0.86 (0.19 to 4.0)	0.85	5.6 (1.3 to 24.3)	0.021
Diagnosis (PM referent)	0.78 (0.24 to 2.54)	0.681	1.14 (0.26 to 5.1)	0.86	0.30 (0.1 to 1.5)	0.141
Sex-diagnosis interaction	6.41 (1.22 to 33.7)	0.028	12.5 (0.65 to 241.5)	0.10	15.7 (1.9 to 128.0)	0.010
IV corticosteroid (vs oral)	-	-	5.6 (1.6 to 20.3)^b^	0.008	-	-
MTX (vs AZA)	-	-	-	-	0.96 (0.3 to 2.8)	0.938

^a^AZA = azathioprine; CK = creatine kinase; IIM = idiopathic inflammatory myopathy; ILD = interstitial lung disease; IV = intravenous; MTX = methotrexate; PM = polymyositis. ^b^Steroid association was restricted to the white subset of the study population when stratified models were used. Inclusion of initial CK level, comorbid ILD or cancer (ever or within 3 years of IIM diagnosis) in additional models did not substantively affect results.

On the basis of univariate Cox regression, age at diagnosis was significantly associated with survival (HR = 1.06, 95% CI = 1.02 to 1.09; *P *= 0.001), indicating that each year of increase in age at diagnosis was associated with a 6% increase in the hazard ratio of death. Race was likewise associated with survival (HR = 1.7, 95% CI = 1.05 to 2.82 for non-Caucasians versus Caucasians). The first CK level and ILD were not associated with survival (HR = 1.0, 95% CI = 1.0 to 1.0, and HR = 0.7, 95% CI = 0.3 to 1.9), respectively.

Multivariable Cox analysis regression was performed to further explore the above-described associations (Table 2). When medication use was excluded, statistically significant associations were found for sex, age at diagnosis, race and interaction between sex and PM and DM diagnoses. When immunosuppressive use was additionally modeled, the results were similar to the model without medication use. Because of nonconformance with the proportional hazards assumption shown in Figure 4, the results from the Cox regression models incorporating immunosuppressive drug type have limited utility. In the multivariable model including steroid use, a statistically significant increase in the HR of death persisted for IV versus oral delivery (HR = 5.6; 95% CI = 1.6 to 20.3). Moreover, estimates for race and the interaction between sex and diagnosis were no longer significant when adjusting for steroid use, and the significance level for the main effect of sex was reduced to borderline. Separate models were consequently fit according to race and revealed that IV versus oral steroid treatment persisted only as a significant predictor for Caucasians, among whom the magnitude of association also became stronger (HR = 10.6, 95% CI = 2.1 to 52.8 Caucasians; HR = 1.3, 95% CI = 0.2 to 11.7 African Americans).

When steroid and immunosuppressive use were simultaneously included in the multivariable model, the results were similar to the multivariable model with steroid but not immunosuppressive use (data not shown). No appreciable differences in effect estimates were found CK level or ILD was added to the models (data not shown). Cancer (defined as either ever or within 3 years of IIM diagnosis) likewise was not included in the final models, because it was not significantly associated with survival and did not materially affect the estimates of other covariates in the models (data not shown).

Causes of death among the 27 patients who died during the follow-up period were cardiac in 22% (4 acute myocardial infarction and 2 congestive heart failure), respiratory in 22% (2 interstitial lung disease, 2 acute respiratory distress syndrome, 1 respiratory failure and 1 asthma), infection in 15% (2 sepsis and 2 pneumonia), cancer in 11% (1 lung, 1 sarcoma and 1 esophageal) and other causes in 30% (1 renal failure, 1 multiorgan failure, 1 brain hemorrhage, 1 intestinal ischemia/bowel infarction, 1 dermatomyositis, 1 traumatic injury and 2 unknown).

## Discussion

The effect of treatment on outcomes of complex diseases such as PM and DM is challenging to study because of factors that include the rarity of these disorders, the heterogeneity of clinical phenotypes and the lack of sensitive outcome measures. There are no large-scale, randomized controlled trials of IIM confirming the efficacy of commonly recommended immunosuppressive drugs. In our study, we evaluated a relatively large cohort of 160 patients and offer a unique perspective on prognostic factors for patients with PM and DM.

Overall 5-year survival in our cohort was 77%, which is consistent with prior estimates of 60% to 80% [5-8], though a small British study reported 95% [13]. Of the 27 deaths, cardiovascular and respiratory disease were the leading causes (22% each), followed by infections and cancer. Autoimmune disorders, such as lupus, are recognized to increase risk of cardiovascular disease, which is not explained by traditional risk factors [14] but by rather adverse effects of cumulative steroid exposure and proposed mechanisms such as endothelial cell apoptosis [15] and increased IFN-α expression [16]. As reviewed by Lundberg and Forbess [17], cardiovascular disease, infection and cancer have been observed to be the leading three causes of death in other myositis studies. The high ranking of respiratory disease as a cause of death in our study could in part be a reflection of our center's serving as a tertiary care referral center for ILD, which could factor into the number or severity of patients with these concomitant diagnoses.

We evaluated whether the initial steroid regimen had an impact on survival and found that patients treated initially with IV corticosteroids had higher mortality that was likely related to disease severity. Of patients receiving pulses of IV steroids, 90% were hospitalized, which may indicate greater perceived disease severity warranting inpatient management. Other factors, such as comorbidities, poor functional status at baseline and complications of high-dose steroid use, could also contribute to an ostensible increase in mortality associated with IV steroid use. Only seven patients (4%) were treated without steroids. We categorized initial steroid exposure as either high to low or moderate dose, and by route, because mechanisms of action may vary accordingly. The molecular mechanisms of glucocorticoids are complex and involve at least four pathways, which were reviewed by Buttgereit *et al*. [18]: the classic genomic action through activation of the cytosolic glucocorticoid receptor (cGR), a secondary nongenomic action initiated by the cGR, a membrane-bound glucocorticoid receptor (mGR)-mediated nongenomic action and nonspecific, nongenomic actions involving cell membranes. High concentrations of glucocorticoids likely cause the nonspecific nongenomic immunosuppressive and anti-inflammatory effects by permeating plasma and mitochondrial membranes and are thought to underlie the rapid effects associated with IV pulse therapy [19]. Though steroids are widely used for the treatment of IIM, the role of corticosteroid dose has not been formally assessed in a large prospective cohort. A retrospective analysis showed that patients treated with high doses of prednisone displayed improved muscle strength and earlier recovery of function [20]. Another retrospective study found that high-dose prednisone regimens offered no survival benefit over low-dose regimens in patients with PM [21].

In our study, the finding of increased mortality in association with IV versus oral steroids was observed among Caucasians, though not among other racial groups. It is unclear why this association was restricted to Caucasians, but varying immunogenetic profiles exist among IIM patients from different racial and ethnic groups and such disease heterogeneity could lead to differential treatment responses [22]. Alternatively, potential differences in prescribing patterns between races could bias results. In our population, 41% of African Americans and 15% of Caucasians received IV corticosteroids (*P *= 0.046). Unfortunately, we are unable to tease apart the factors underlying the choice of corticosteroid regimen. The proportion of patients with comorbid ILD was not statistically different between Caucasians (21%) and African Americans (36%), whereas CK levels were higher among African Americans (median = 1,256, IQR = 257 to 7,727) compared with Caucasians (median = 333, IQR = 94 to 1998) (*P *= 0.02). Retrospective categorization of disease severity is problematic for inflammatory myopathies because of inconsistencies in muscle-strength assessment and other nonstandard measures of disease activity.

In our study, we compared the two most commonly prescribed immunosuppressive medications, AZA and MTX, both of which are used widely for the treatment of autoimmune diseases as steroid-sparing agents. An open-label trial of 22 patients with refractory disease showed that placing patients on MTX reduced the daily prednisone dose at 6 months [23]. In a randomized controlled trial designed to assess the effect of AZA in combination with prednisone versus prednisone alone, 16 patients with PM were studied. There was no significant difference between the two groups after 3 months [24], but the 3-year follow-up showed an improvement in function and a decrease in daily prednisone dosage [25].

MTX and AZA have been compared in only one double-blinded randomized controlled trial of 28 patients with PM and DM [26]. Handheld myometry was the primary end point, which is a measure of grip strength that has not been validated for patients with inflammatory myopathies, and testing of proximal muscle groups may be more sensitive to change. The hand-grip strength after 1 year was similar between MTX and AZA groups, although MTX was better tolerated.

In our study, as depicted in the unadjusted Kaplan-Meier curves, 5-year survival was similar among patients treated with MTX and those treated with AZA, whereas survival was higher in the MTX group from 5 years onward. In the Cox model, adjusting for various demographic and clinical factors, a difference in the hazard ratio of mortality according to type of immunosuppressive was not detected, though because of the lack of conformity with the proportional hazards assumption, these results must be interpreted with caution. Our results are compatible with those from a cohort of 113 patients (PM, DM and IBM) suggesting superiority of MTX versus AZA in terms of complete and partial clinical responses (subjectively defined) among men, as well as anti-synthetase antibody-positive subsets [27]. In our study population, baseline characteristics were similar among patients treated with MTX versus AZA, including CK levels and ILD, which are potential indicators of disease severity. In fact, though not reaching a statistically significant difference, CK levels were higher in the MTX group. Although the presence of lung disease could influence the selection of immunosuppressive agents, with AZA potentially being chosen for ILD patients to avoid MTX-associated lung injury, we were not able to retrospectively confirm a rationale for choice of drug. However, the proportion of ILD patients treated with AZA (26%) was similar to those who were given MTX (21%) (*P *= 0.586). In this PM and DM population, we also did not find an association between comorbid ILD and survival.

Several studies have suggested that higher CK levels are associated with increased disease activity [11,13,28], though levels are not always reliable as predictors of disease activity or therapeutic response, despite their being used widely in clinical practice. A higher initial CK level may be associated with a more rapidly progressing course, prompting patients to seek treatment more quickly. In this cohort, initial CK was not associated with survival. Similarly, though the initial CK level was significantly higher for PM than for DM, there was no significant difference in survival between these groups. The higher CK levels in the IV pulse treatment group did not reach statistical significance compared with the other two treatment groups.

In our study, we found a female-to-male ratio consistent with other studies. Ten-year survival was significantly lower among males versus females, with the disparity being most evident in the PM group. These data are consistent with accumulating data from studies of other autoimmune diseases, such as lupus, suggesting greater severity among men despite higher incidence among women [29]. As reviewed elsewhere, however, the results from prior myositis studies are inconsistent with respect to whether male sex is associated with reduced survival [17].

Several limitations of the present study should be considered, including its retrospective nature and conduct at a single center. Though it would be interesting to assess survival from disease onset and the potential impact of delay until diagnosis, we were unable to do so because of the difficulty in reliably ascertaining dates of IIM onset. Detailed autoantibody profiles were likewise not uniformly available, though such data would be of prognostic interest. Our center is a tertiary care facility, and it is possible that our cohort could have had greater disease severity due to referral bias. Although IV pulse steroid treatment was found to be associated with high mortality in this population, it is likely that this observation is due to confounding by indication whereby patients may have received this treatment because of more severe disease. We could not determine the reasons underlying the choice of immunosuppressive regimen, but the apparent difference in long-term survival favoring MTX may be attributable to confounding factors and therefore may not represent a true difference between the two treatment groups.

## Conclusions

In summary, we have identified variables associated with survival in a large cohort of patients with PM and DM. We were specifically interested in determining the comparative effectiveness of different regimens of corticosteroids and immunosuppressive drugs. We could not identify improved survival associated with the use of pulsed IV corticosteroids, and, in fact, survival was lower, which was likely related to disease severity. Survival at 5 years was similar for patients treated with MTX and AZA, and the observed higher survival associated with MTX after 5 years could not be confirmed in multivariable modeling for the full follow-up period. Our data underscore the importance of evaluating long-term outcomes in the real-world setting, and prospective, multicenter studies should be conducted to further explore these findings.

## Abbreviations

AZA: azathioprine; CK: creatine kinase; DM: dermatomyositis; IBM: inclusion body myositis; IFN: interferon IIM: idiopathic inflammatory myopathy; ILD: interstitial lung disease; IV: intravenous; MTX: methotrexate; PM: polymyositis; TNF: tumor necrosis factor.

## Competing interests

The authors declare that they have no competing interests.

## Authors' contributions

ES and KP contributed to the study design, performed data interpretation and drafted the manuscript. PM participated in the study design, performed data collection and assisted in interpretation of results and review of the manuscript. LJC conceived the study, oversaw the study design and its coordination, and contributed significantly to data interpretation and drafting the manuscript. ECS participated in the study design and its coordination, conducted statistical analysis and data interpretation, and participated in drafting the manuscript. All authors read and approved the final manuscript for publication.
